# The Initial Months of COVID-19: Dog Owners' Veterinary-Related Concerns

**DOI:** 10.3389/fvets.2021.629121

**Published:** 2021-02-02

**Authors:** Lori R. Kogan, Phyllis Erdman, Cori Bussolari, Jennifer Currin-McCulloch, Wendy Packman

**Affiliations:** ^1^Department of Clinical Sciences, Colorado State University, Fort Collins, CO, United States; ^2^College of Education, Washington State University, Pullman, WA, United States; ^3^Department of Counseling Psychology, University of San Francisco, San Francisco, CA, United States; ^4^School of Social Work, Colorado State University, Fort Collins, CO, United States; ^5^Department of Psychology, Palo Alto University, Palo Alto, CA, United States

**Keywords:** COVID-19, dog, veterinary, protocol, veterinary care, human animal bond

## Abstract

Veterinarians, like many other professions, were significantly impacted by the onset of COVID-19 in the spring of 2020. Standard practices were disrupted, and veterinary hospitals had to quickly modify standard protocols to safely serve their clients and patients. The purpose of this study was to better understand dog owners' fears and concerns pertaining to veterinary care and obtainment of pet care products and food during the lock down phase of a pandemic to be better prepared to address these concerns now and in the future. To this end, an online, anonymous, cross-sectional survey was designed and distributed to adult dog owners via social media. The results, from a total of 4,105 participants (the majority from the United States and Canada), indicated substantial areas of concern. The number one concern of dog owners during this time was the availability of emergency veterinary care. Owners under 30 years of age, compared to older owners, were significantly more concerned about both availability and cost of veterinary care (emergency and non-emergency). The ability to care for one's dog if they were to become ill was a concern for many owners, yet only 60% had identified a caretaker for their dog if one was needed. These results suggest that the majority of dog owners remained true steadfast guardians of their dogs, continuing to make them a priority, even during pandemic times. Suggestions to help mitigate dog owners' concerns and improve communication between owners and veterinarian teams are offered.

## Introduction

The American Veterinary Medical Association (AVMA) disseminated a survey in April 2020 to US veterinary practice owners to better understand how COVID-19 had affected their practice (1). The survey included questions about operational changes, client numbers, use of personal protective equipment, and financial impact—and found that all of these areas were significantly impacted by COVID-19. One of the areas most impacted was daily operations. Responding to the need for social distancing, most veterinary hospitals implemented curb side care, with clients waiting in their vehicles while their pet was treated inside. Many hospitals were forced to cancel or limit their appointments and new protocols (and resultant expense) related to the increased need for sanitation and personal protective equipment (PPE) were implemented. The initial financial repercussions were significant, in part due to dramatic decreases in client visits, which resulted in substantial cash shortages. The sudden and serious onset and impact of COVID-19 left pet owners and veterinary professionals alike struggling to find reliable scientific information about the pandemic and its possible impact on humans and animals alike.

The rapid new findings related to COVID-19 and the efforts to obtain and report accurate, reliable information is ongoing. The Center for Disease Control and Prevention (CDC) published the *Interim Infection Prevention and Control Guidance for Veterinary Clinics Treating Companion Animals* (2) in April 2020 and has continued to update it several times. The report is intended to help veterinary clinics “facilitate preparedness and ensure that practices are in place to help people and animals stay safe and healthy.” The information is focused on how veterinarians can adjust their protocols in regards to optimum human safety. There is no mention of pet owners' concerns.

Another recent survey assessed how emergency veterinary hospitals were responding to COVID-19 (3). Even with the CDC's suggestions regarding procedural strategies (2), this study found significant variation in how veterinary hospitals interpreted and implemented these suggested policies. Some hospitals continued to provide emergency care, but others that were not able to provide care referred cases back to the owners' primary care veterinarian or provided minimal treatment with additional monitoring by the owner. These hospitals also reported dealing with issues of safety protocols, shortages of staff, and financial impact.

Although both of these surveys (1, 3) documented important information that remains critical in adjusting veterinary care to meet client and patient needs during a pandemic, the only item on either of these surveys pertaining to clients' COVID-19 related concerns was about the possible transmission of COVID-19 between humans and animals in the AVMA survey. The impact of the necessary procedural changes on clients (e.g., curb side care, canceling appointments, limiting surgeries) was not addressed. Likewise, questions such as “What do I do if I have an emergency with my pet? What if I can't find a veterinarian open? What if my pet needs special medication and I cannot access veterinary care?” were left unanswered.

One of the first studies pertaining to veterinary clients' perspective was conducted by Banfield Pet Hospital (4). In this study, a sample of 1,000 dog and cat owners responded to a range of questions regarding how their personal well-being and concerns about their pets' health has been impacted by COVID-19. Eighty-four percent of respondents reported feeling more attuned to their pet's health, and 37% report paying more attention to their pet's care. Other recent studies have reported similar concerns in the United States as well as Spain and the United Kingdom (5–7).

These findings, in addition to the changes within the veterinary field, suggest that COVID-19 has impacted pet owners in many critical areas, yet the subject remains unexplored. Understanding the impact of COVID-19 on pet owners can help veterinary professionals best address their clients' concerns and needs during a pandemic.

## Materials and Methods

### Procedures

The purpose of our survey was to better understand dog owners' fears and concerns pertaining to veterinary care and obtainment of pet care products and food during the lock down phase of a pandemic to be better prepared to address these concerns now and in the future. Because dog owners may have unique concerns, in comparison with other companion animals, the decision was made to limit this study to dog owners. A subsequent separate study was initiated for cat owners. To this end, we developed an online, anonymous, cross-sectional survey using Qualtrics (Qualtrics, Inc., Provo, UT, USA). The survey was designed, reviewed, and tested by the co-investigators and their colleagues (veterinary and social science professionals) after seeking input from dog-owning representatives from the community. The survey was pilot tested for ambiguity and/or potentially missing or inappropriate response options, with revisions made based on the results of the pilot testing. The final survey and study design were approved by the Colorado State University Institutional Review Board (IRB # 20-10003H). Survey respondents were recruited through social media outlets and human animal focused organizations (e.g., Facebook dog focused groups, Human Animal Interaction Section of American Psychological Association, etc.) between March 30, 2020 – May 1, 2020.

### Description of Study and Participants

In order to maximize our understanding of the impact of COVID-19, dog owners at least 18 years of age from any country were encouraged to participate with the recognition that countries' veterinary services (within countries as well as between) might be impacted differently. Demographic data were collected (e.g., age group, gender, country of residence, current level of COVID-19 restrictions, number of adults, children and dogs living in the home). Respondents were asked to indicate their social support before COVID-19 and at the time they completed the survey and the psychological impact of having dogs during COVID-19 restrictions (analyses of these items are not included in this paper). Additionally, they were asked about changes in how much time they spent with their dog (in general, actively engaged, and walking). Next, they were asked about their perception of any potential changes in the bond they have with their dog before COVID-19 and currently with two bond rating questions that asked them to rate their bond with their dog on a scale from 1 (not at all bonded) to 10 (extremely bonded). Potential changes in owners' perceptions of dog behaviors were assessed with two questions. The first question asked owners to indicate, 1 month before the COVID-19 outbreak, how frustrated they felt about their dogs' undesirable behaviors on a scale from 1 (minimal/no frustration) to 10 (extremely frustrated). They could also select “no undesirable behaviors.” This question was followed with a similar question asking owners to rate their current level of frustration.

The next section of the survey included questions related to veterinary care and non-veterinary dog related concerns using a 4-point Likert scale (no concern to great concern). Veterinary-related questions included level of concern about their ability to afford veterinary care (emergency and non-emergency both now and in the future) and concern about the availability of their veterinarian for emergency and non-emergency issues. The next set of questions pertained to dog supplies (affordability and access), caretaker concerns, and zoonotic risks. These included the ability to afford or acquire dog food/supplies now or in the future, a caretaker for their dog if they themselves became ill or were unable to play/exercise with them, and whether the virus could be passed between dog and owner. They were also asked to indicate their concern about their ability to keep their dog due to COVID-19 changes.

Owners were asked if they had designated a caretaker in case they were unable to care for their dog. The next set of questions asked about their veterinary care experiences during COVID-19 restrictions. Lastly, we asked about their past experience and future plans for volunteering for an animal shelter/rescue organization.

Data were analyzed using SPSS Version 25 (IBM, Armonk, NY, USA). Descriptive statistics were calculated to characterize current attitudes and patterns in dog care during COVID-19 restriction times. Paired *t*-tests were used to assess changes in bond score and frustration level with behaviors from 1 month before COVID-19 to “current” time. Associations between demographic variables and concern related questions were investigated using Kruskal-Wallis nonparametric analysis of variance. Significance level (α) was set at *p* = 0.05 and all tests were two-tailed.

## Results

The total number of responses to the survey was 4,105. At the time of this survey, most participants reported restrictions in their city at the level of “all non-essential stores/businesses closed and ordered/strongly recommended to stay at home” (3,197, 77.9%) followed by “all non-essential stores/businesses closed but no order to stay at home” (724, 17.5%). A small number reported “some stores/businesses closed” (151, 3.7%) or “no restrictions” (17, 0.4%) or “other” (1, 0.4%). Most participants were from the US (3,313, 80.7%); followed by Canada (355, 8.7%), UK (177, 4.3%), and Australia (64, 1.6%); 194 (4.7%) were from other countries including Brazil (29, 0.7%), Germany (12, 0.3%), Ireland (12, 0.3%), Norway (21, 0.5%), South Africa (12, 0.3%) and <10 responses from a wide array of other countries.

When queried about number of people and dogs in the home, the most common response for number of adults in the home was two (2,533, 61.7%), followed by one (872, 21.2%). The majority of participants did not have children under the age of 18 living at home (3,305, 80.5%), followed by one child (423, 10.3%). Most participants owned one dog (2,028, 49.4%) with many others reporting owning two dogs (1,286, 31.3%) or three dogs (479, 11.7%).

The age range of participants (*n* = 4,058) was 18–92 [Mean = 48.06 (SD 14.27), Median = 49]. For analysis, ages were categorized into younger than 30 (558, 13.8%), 30–39 (772, 19.0%), 40–49 (790, 19.5%), 50–59 (966, 23.8%), and 60 and older (972, 24.0%). The majority of respondents were female (3,610, 88.3%), with 443 (10.8%) males and 34 (0.8%) identifying as non-binary (18 did not answer).

The survey questions pertained to both the bonding activities with their dogs, as well as veterinary-related concerns. In this article we are only presenting the veterinary-related concerns; however, it is important to note that 72.1% of respondents indicated they spent more time with their dogs, 64.3% reported increased time spent actively engaged with their dogs, and 55.2% reported that they felt a strengthened bond with their dog.

### Veterinary Related Issues

#### Potential Availability of Emergency and Non-emergency Care

Participants were asked to indicate their level of concern with several veterinary issues (*n* = 3,996). The issues of most concern were whether their veterinarian would be available if they needed them for emergencies and non-emergencies (Table 1). Participants under 30 years of age, compared to older owners, were significantly more concerned about their ability to afford emergency veterinary care in the future and non-emergency veterinary care now. They were also more concerned that their veterinarian will not be available if they needed him/her for both non-emergencies and emergencies. Additionally, the increased amount of concern by owners under 30 years of age regarding their current ability to afford emergency care, when compared to older owners, approached statistical significance (*p* = 0.059).

**Table 1:** COVID-19 related concerns for veterinary care and dog food/supplies and the impact of owners' age.

	**Great concern**	**Some concern**	**No/minimal concern**	**NA/not an issue**	**H (df) *p*-value**
Concern that my vet will not be available if I need them for emergencies^*^	898 (22.5)	1,544 (38.9)	1,458 (36.5)	96 (2.4)	11.46 (4), *p* = 0.022
Concern that my vet will not be available if I need them for non-emergencies^*^	672 (16.8)	1,434 (35.9)	1,786 (44.7)	104 (2.6)	13.29 (4), *p* = 0.010
Ability to afford emergency veterinary care now	521 (13.0)	1,153 (28.9)	2,114 (52.9%)	208 (5.2)	9.09 (4), *p* = 0.059
Ability to afford emergency veterinary care in the future^*^	569 (14.2)	1,231 (30.8)	2,026 (50.7)	170 (4.3)	10.41 (4), *p* = 0.034
Ability to afford non-emergency veterinary care now^*^	245 (6.1)	807 (20.2)	2,746 (68.7)	198 (5.0)	10.17 (4), *p* = 0.038
Ability to afford non-emergency veterinary care in the future	279 (7.0)	939 (23.5)	2,610 (65.3)	168 (4.2)	7.83 (4), *p* = 0.098
Ability to afford dog food/supplies now^*^	100 (2.5)	542 (13.6)	3,197 (80.0)	157 (3.9)	19.78 (4), *p* = 0.001
Ability to afford dog food/supplies in the future^*^	170 (4.3)	716 (17.9)	2,976 (74.5)	134 (3.4)	12.29 (4), *p* = 0.015
Ability to obtain (not due to financial reasons) dog food/supplies	389 (9.7)	1,341 (33.6)	2,175 (54.4)	91 (2.3)	5.15 (4), *p* = 0.272

* *Statistically significant age difference*.

#### Care and Feeding

Participants were also asked to indicate their concern level with several animal, non-veterinary-related issues (*n* = 3,996). The areas of most concern were the ability to play/exercise their dog or obtain a caretaker for their dog if they contracted COVID-19 (Table 2).

**Table 2:** COVID-19 related concerns for non-veterinary dog issues and the impact of age on concern level.

	**Great concern**	**Some concern**	**No/minimal concern**	**NA/not an issue**	**H (df), *p*-value**
Ability to exercise/play with your dog if you get COVID-19^*^	974 (24.4)	1,413 (35.4)	1,525 (38.2)	83 (2.1)	26.06 (4), *p* < 0.001
Concern about having to leave the house if my dog gets injured or sick	552 (13.8)	1,076 (26.9)	2,218 (55.5)	150 (3.8)	3.08 (4), *p* = 0.545
Ability to keep your dog because of changes due to COVID-19^*^	461 (11.5)	539 (13.5)	2,803 (70.2)	192 (4.8)	79.20 (4), *p* < 0.001
A caretaker for your dog if you contracted COVID-19^*^	866 (21.7)	1,124 (28.1)	1,817 (45.5)	189 (4.7)	50.50 (4), *p* < 0.001
Concern that you could give your dog COVID-19^*^	191 (4.8)	423 (10.6)	3,215 (80.5)	167 (4.2)	14.51 (4), *p* = 0.006
Concern that your dog could give you COVID-19^*^	46 (1.2)	176 (4.4)	3,608 (90.3)	166 (4.2)	17.93 (4), *p* = 0.001

* *Statistically significant age difference*.

Participants under 30 years of age, compared to older owners, reported more concern about their ability to afford dog food/supplies now and in the future, and the zoonotic risks of COVID-19 (passed from owner to dog and from dog to owner). Participants 60 years of age and older reported higher concern, compared to younger owners, about acquiring a caretaker for their dog if they contracted COVID-19 and the ability to keep their dog because of COVID-19 related changes. The ability to exercise/play with their dog if they contracted COVID-19 was of high concern for participants 18–29 years old and participants 60 years of age and older when compared to participants between the ages of 30–59.

Participants were asked if they had identified someone who could care for their dog if they, as owners, became sick (*n* = 3,969), to which 2,357 (59.4%) reported yes. Participants 50 years of age and older were more likely to report identifying a caregiver, compared to younger owners [X^2^ = 60.69 (4), *p* < 0.001]. A caretaker was identified by 55.4% (299) of owners 29 years and younger, 52.2% (391) by owners 30–39 years of age, 54.0% (409) by owners 40–49 years of age, 62.9% (589) by owners 50–59 years of age, and 67.8% (639) by owners 60 and older. When asked if they had agreed to care for someone else's dog if that owner were to become ill (*n* = 3,969), 643 (16.2%) reported “yes,” 3,124 (78.7%) reported “no” because they had not been asked, and 92 (2.3%) reported “no,” they had been asked, but could not commit to this responsibility. Participants 50 years of age and older, when compared to younger owners, were more likely to report agreeing to care for someone else's dog, [X^2^ = 33.53 (12), *p* = 0.001].

#### Use of Veterinary Services

Participants were asked if their dog had required veterinary care since the COVID-19 outbreak to which 887 (22.3%) reported yes. Of those who said they required veterinary care, the majority reported taking their dog to a veterinarian (709, 79.8%). Those who reported going to the veterinarian were asked to indicate all the reasons for the veterinary visit(s). The most common reasons were vaccinations, monitoring an illness/disease or wellness exam. The numbers for each type of visit are listed in Table 3.

**Table 3:** Reasons for veterinary visits since the COVID-19 outbreak reported by 4,103 dog owners.

**Reasons**	**Total**	**US (*n* = 3,313)**	**Canada (*n* = 355)**	**UK (*n* = 177)**	**Australia (*n* = 64)**	**Other (*n* = 194)**
Vaccinations	193 (4.7%)	177 (5.3%)	12 (3.4%)	1 (0.6%)	2 (3.1%)	1 (0.5%)
Wellness exam	167 (4.1%)	158 (4.8%)	6 (1.7%)	2 (1.1%)	0	1 (0.5%)
Monitoring an illness/disease	162 (3.9%)	138 (4.2%)	8 (2.3%)	7 (4.0%)	4 (6.3%)	5 (2.6%)
Skin/allergy	93 (2.3%)	82 (2.5%)	6 (1.7%)	3 (1.7%)	2 (3.1%)	0
Vomiting/diarrhea	68 (1.7%)	58 (1.8%)	5 (1.4%)	4 (2.3%)	0	1 (0.5%)
Surgery	48 (1.2%)	42 (1.3%)	3 (0.8%)	0	0	3 (1.5%)
Serious illness (e.g., cancer)	42 (1.0%)	37 (1.1%)	2 (0.6%)	1 (0.6%)	0	2 (1.0%)
Dental	37 (0.9%)	34 (1.0%)	1 (0.3%)	2 (1.1%)	0	0
Ear/eye	14 (0.3%)	10 (3.0%)	2 (5.6%)	0	2 (3.1%)	0
Accident	38 (0.9%)	28 (0.8%)	4 (1.1%)	0	4 (6.3%)	2 (1.0%)
Euthanasia	10 (0.2%)	10 (0.3%)	0	0	0	0
Other	57 (1.4%)	36 (1.1%)	14 (3.9%)	3 (1.7%)	2 (3.1%)	2 (1.0%)

#### Veterinary-Client Interactions

Participants were asked about the procedures in place when they visited their veterinarian (*n* = 696). The largest percent reported they were met in the parking lot and not allowed inside the hospital (398, 57.2%), followed by allowed in both the reception area and exam room (179, 25.7%) and then allowed in reception area only (51, 7.3%). Most of the “other” responses (68, 9.8%) reported that the protocols changed over time and they had experienced more than one protocol.

Lastly, participants were asked to describe their most recent veterinary visit experience since the COVID-19 outbreak, during a time where, in most instances, humans were not allowed in the clinics. Several pertinent issues emerged. The most prominent theme reflected participants' concerns regarding their inability to be present with their dog in the veterinary clinic. This was most evident during a euthanasia situation, for example, “Had to put dog down. Sad that I could not hold her. She died alone.” Dog guardians felt the need to be with their dog to support and take care of them and experienced stress as a result, even with a procedure as benign as a nail trim. One participant noted, “(Vet) is great because she communicates so well via email and I know that (dog) loves going there. She even texted pictures during this acupuncture appointment.” Other concerns revolved around participants' own worries about their dog's physical and emotional well-being: “Waited in car while vet took my dog from my car to the clinic. Dog experienced greater anxiety with this protocol.” Importantly, some of the more intense concerns were mitigated by the attention, responsiveness, communication and transparency of the veterinary staff. For instance, “Put my heart dog to sleep and had a wonderful support by my vets.” Many participants were appreciative of their veterinarian's communication pertaining to COVID-19 transmission risks and the extra safety precautions their hospitals implemented: “The receptionist invited us into the clinic while reassuring me that everything had just been sterilized. I was very impressed with their professionalism.” Another participant commented: “Pleasant and good rapport with veterinarian. She provides good care and answers questions pertaining [to] Corona spread concerns humans and animals. We both understand risk of exposure between her and I.” Conversely, some participants noted they felt stressed by the imposed restrictions. One participant noted: “[I] did not like it [veterinary visit] because I could not interact with the vet face-to-face and tell him my concerns. I had to tell the technician and forgot one.” Overall, it seems that the interpersonal engagement of the veterinary clinic, with both clients and patients, impacted the perception of overall experience.

## Discussion

This study was conducted during the initial months of the COVID-19 pandemic, when the majority of people were in “lock-down” mode, meaning that they were instructed or at least strongly recommended to stay at home and all non-essential stores/businesses closed. This situation was unprecedented, resulting in a great deal of anxiety and uncertainly about the future. This anxiety encompassed all areas of daily living, so it is not surprising that dog owners felt significant concerns related to providing for their dog, nor the fact that these concerns have been expressed by other dog owners in the United States as well as Spain and the United Kingdom (5–7).

It is noteworthy that the top concern of dog owners during this time was the availability of their veterinarian for both emergency as well as non-emergency care. This concern was felt most strongly by dog owners 18–29 years of age. Even though veterinary services were classified as essential and therefore permitted to remain operating, it appears this did little to assuage dog owners' fears. These first months were a time in which veterinary hospitals were quickly developing new protocols during a time of limited COVID-19-related information and knowledge. Certainly, these owners' concerns were not unfounded, as veterinary clinics struggled with how to safely offer care. It took hospitals time to develop what is currently being utilized by most veterinary clinics—“curb side” care—in which clients remain outside in their cars while their pets are treated inside. Although far from ideal, this protocol has allowed veterinary clinics to continue operating with limited human to human contact. Perhaps because younger owners have grown up in a time period when nearly all services and products are consistently and reliably available (and almost instantly), they were more impacted by these initial uncertain times.

One lesson that might be gleaned from these initial months and owners' fears is the need for communication. Results from this study further document the invaluable relationship these respondents have with their dog(s) and, hence, how important it is for veterinarians to understand this relationship and the need for owners to be assured they have access to veterinary care during these times, and that their dogs will be well cared for, regardless of COVID-19 restrictions. While hospitals were struggling to implement new protocols, it is unknown how many of them were regularly reaching out to keep their clients informed. Communication of this type does not have to be overly complicated or time consuming but should utilize several communication modes. For example, the use of veterinary clinic mobile apps, text messages and Instagram might be best suited for communicating with younger pet owners, while Facebook and phone messages might work better with older clients. Sending frequent messages, regardless of modality, might help decrease owners' concerns regarding veterinary clinics' availability. As COVID-19 cases continue to rise, or future instances of radical change that necessitate societal restrictions occur, this need to ease owners' concerns will likely only increase.

Another major area of concern reported by dog owners during this time was the ability to afford veterinary care and dog supplies. Again, these fears were most pronounced for dog owners younger than 30 years of age, which contradicts research that has suggested that younger pet owners are more willing to pay higher costs for veterinary services when compared to owners 50 and older (9). It is possible that these results are due to the employment impact of COVID-19 felt by younger Americans.

While COVID-19 resulted in large numbers of people losing their jobs, the impact disproportionally affected younger workers, many of whom work in service sector jobs (i.e., retail, food and drinking establishments) (8). Strategies to help owners afford the care they need for their dogs include the offering of wellness plans, CareCredit, and pet insurance. Owners who are concerned about affording pet supplies could be directed to local pet food banks/pantries. Communicating and publicizing these local services can help clients feel their veterinary hospitals are concerned about all aspects of their pets' well-being.

Another aspect of pets' care pertained to plans for one's dog if the owner became ill. Owners expressed considerable concern about how they would be able to play with and exercise their dog if they became ill. This concern was felt most by owners younger than 30 years of age as well as those 60 years and older. A related concern was the ability to obtain a caregiver for their dog if they became ill. This concern was stronger for older owners compared to younger owners. While veterinary clinics cannot directly provide services to meet these needs, acknowledging and validating these fears is important and could be conveyed through blogging about these topics as well providing information about available resources (e.g., dog day care, dog walkers, etc.). Areas of less concern were owners' fears of giving or contracting COVID-19 to/from their dog. This finding is encouraging, suggesting that owners were able to access trustworthy news sources and were able to receive accurate information about these potential risks.

It is critical to note that although many owners express concerns related to caring for their dog if they become ill, only 60% had identified a caretaker. Even for owners 60 and older, this percentage (68%) still leaves a considerable number of dogs at potential risk. In addition, the majority (78%) indicated they had not been asked to care for someone else's dog, yet it was higher for participants 50 years of age and older when compared to younger owners. Perhaps this is due to younger owners having children in the home, other responsibilities or being away from home more than older dog owners. The reasons for these age differences warrants further study. Veterinary teams can positively impact both owners and their dogs by proactively asking owners if they have designated a caretaker. This could be as simple as adding a question to the intake/admission form (and explaining the importance of doing so). In this way, it does not have to take any additional appointment time. In terms of veterinary care during the initial months of COVID-19, it is noteworthy that even with all these additional stressors and distractors people experienced during this time, nearly 25% said they needed veterinary care and the majority (80%) of these people reported visiting their veterinarian to get the care they needed. The fact that this percentage varied among countries (e.g., from 24% in the US to 13% in the UK) is worthy of future study.

## Implications for Further Research

In summary, this study has limitations inherent in online surveys including the potential bias of those who chose to participate. It was only available to dog owners who had access to the internet and the participants were largely female, so care should be taken when generalizing to other populations. This study was also completed during the beginning months of COVID-19, so we do not know how the continued restrictions have impacted peoples' lives and experiences with their dog. Future research could include a longitudinal design to see how people's experiences have evolved over time. We also did not include veterinarians, so a survey, or interviews, that included veterinarians could give insight into some of the challenges they have faced in responding to their clients' needs. A factor that was not considered was income level. Owners with lower incomes have fewer resources and/or options for pet veterinary care and supplies. This is likely exacerbated by the pandemic, only adding more stress for pet owners.

For the surveyed population however, the results suggest that these dog owners remained true steadfast “guardians” (defined as “someone who defends and protects”) of their dogs. During the first months of COVID-19, while the world was in turmoil and the future uncertain, the care and well-being of their dogs remained a high priority. As the pandemic unfolds, it is hoped that veterinary hospitals continue to evolve their protocols and communication techniques to best partner with dog owners; thereby serving the needs of these guardians and their companions.

## Summary

Veterinarians, like many other professions, were significantly impacted by the onset of COVID-19 in the spring of 2020. Standard practices such as routine animal checkups and surgeries were disrupted, and veterinary clinics and hospitals had to quickly modify standard protocols to safely serve their clients and patients. The purpose of this article is to present the findings of a survey given to dog owners during the initial lockdown phase of COVID-19 to better understand dog owners' veterinary related concerns during this time. Implications and suggestions for the veterinary field, as well as suggestions for future research, are discussed.

## Data Availability Statement

The raw data supporting the conclusions of this article will be made available by the authors, without undue reservation.

## Ethics Statement

The studies involving human participants were reviewed and approved by Colorado State University Institutional Review Board. Written informed consent for participation was not required for this study in accordance with the national legislation and the institutional requirements.

## Author Contributions

LK and PE conceived the study. LK, PE, JC-M, CB, and WP conducted the research and wrote the manuscript. All authors contributed to the article and approved the submitted version.

## Conflict of Interest

The authors declare that the research was conducted in the absence of any commercial or financial relationships that could be construed as a potential conflict of interest.

## References

[1] American Veterinary Medical Association COVID-19 Impact on Veterinary Practices. (2020). Available online at: https://www.avma.org/resources-tools/animal-health-and-welfare/covid-19/covid-19-impact-veterinary-practices (accessed November 7, 2020).

[2] Centers for Disease Control and Prevention Communities, Schools, Workplaces, & Events. (2020). Available online at: https://www.cdc.gov/coronavirus/2019-ncov/community/veterinarians.html (accessed November 7, 2020).

[3] Wayne A Rozanski E. The evolving response by emergency veterinary hospitals during the COVID-19 pandemic. J Vet Emerg Crit Care. (2020). 10.1111/vec.12995. [Epub ahead of print]. 32864824

[4] Today's Veterinary Practice Banfield Study Shows Shifting Views on Pet Healthcare During Pandemic. (2020). Available online at: https://todaysveterinarypractice.com/banfield-pet-owner-covid-19-survey/ (accessed November 7, 2020).

[5] Applebaum JW Tomlinson CA Matijczak A McDonald SE Zsembik BA. The Concerns, Difficulties, and Stressors of Caring for Pets during COVID-19: Results from a Large Survey of U.S. Pet Owners. Animals. (2020) 10:1882. 10.3390/ani10101882 33076475 PMC7602525

[6] Bowen J García E Darder P Argüelles J Fatjó J. The effects of the Spanish COVID-19 lockdown on people, their pets, and the human-animal bond. J Vet Behav. (2020) 40:75–91. 10.1016/j.jveb.2020.05.013 32837452 PMC7292953

[7] Anon. The Impact of COVID-19 Lockdown Restrictions on Dogs & Dog Owners in the UK. Available online at: https://www.dogstrust.org.uk/help-advice/research/research-papers/201020_covid%20report_v8.pdf (accessed December 25, 2020).

[8] Pew Research Center Young Workers Likely to be Hard Hit as COVID-19 Strikes a Blow to Restaurants and Other Service Sector Jobs. Pew Research Center. (2020). Available online at: https://www.pewresearch.org/fact-tank/2020/03/27/young-workers-likely-to-be-hard-hit-as-covid-19-strikes-a-blow-to-restaurants-and-other-service-sector-jobs/ (accessed November 7, 2020).

[9] Felsted KE The Price is Right. Today's Veterinary Business. (2019). Available online at: https://todaysveterinarybusiness.com/the-price-is-right/ (accessed December 29, 2020).

